# Sceptical Doctors

**Published:** 1887-09-24

**Authors:** 


### SCEPTICAL DOCTORS.

"ORTHODOXY, my Lord," said Bishop Warburton, "is my doxy—heterodoxy is another man's doxy." No set of principles or rules formulated by man can ever be absolutely and unquestionably final. There will always be a place for the sceptic, the questioner, the man of new standpoints, the fearless analyst. That which is established should never be assailed for the mere pleasure of conflict; but every man comes into the world with a personality different from that of every other man, and has the power of throwing a kind of light on the problems of life which is peculiar to himself. It is his duty, so far as opportunity offers or can be made, to examine for himself, honestly and thoroughly, every subject on which he is called upon to affirm a judgment or to put forth his hand in action. It is impossible to imagine how dreary the world would be to a thinker if there were no conflict of opinion. By thinker is not meant the mere writer of books or newspaper articles, but the man of independent mind, whatever his occupation or degree of culture, or no culture. There is many a hedger and ditcher who is an incomparably better thinker than some of his fellows who construct elementary grammars for his childhood, or write magazine articles for his guidance and enlightenment when he comes to riper years. There can be no more foolish idea than this, that a man can only think with a pen in his hand. What did men do before pens were made, and in still remoter times before the art of writing was invented? They thought and they acted; they conquered countries and established dynasties; they wooed and married; they sorrowed for the suffering; they wept for the dead; they looked into the dread unknown and hoped for the dawn of an immortal day.

Medicine has not until recent times been famous for its heresies. Too seldom has there arisen a man of intellectual force and honesty who has dared to assail the orthodoxy of his period and to affirm the worth of individual labours. As if conscious of the mental torpidity of the past, the toilers of the present are making all possible efforts to question and deny the value of the results of years of patient labour, and of the stores of accumulated experience.

We admit to the full their right to question; but when they pass from doubt to affirmation, we in turn claim our right to doubt the pertinency of their doubts and to deny the validity of their denials. The result of the doubting of many modern medical men is a denial of the value of most of the medical treatment of the day. Their studies in the fields of theory and practice have led some of them to what Carlyle calls, in another region of thought, the "Centre of Indifference," and others to the affirmation of what he calls "The Everlasting No." Many of them have passed from the stage of uncertainty and ended by the affirmation of a negative. To the question, "Is the orthodox practice of medicine of the present day scientific in principle and effectual in results?" They will unhesitatingly answer "No!" Are they right?

The answer to this question will depend upon what a man understands to be the aim of medical practice. If he is always expecting to "cure" the diseases of the patients he attends: if he sets to work to "cut them short"; to expel them from the system; to regard them as something foreign which must be killed or got rid of in some way or other, then indeed he will often find his principles founded on sand and his practices ineffectual. Then an attitude of scepticism and even utter denial is a tribute at once to his mental acuteness and to his moral rectitude. But is that a correct description of the aim and work of the modern medical man? We venture to say it is not. The *rôle* of the miracle worker is not for the doctor, but the much more apparently humble avocation of the helper and the educated "attendant." There is a wonderful fitness in the term which is universally applied, as if by accident and yet by general consent, to the physician. He is the "medical attendant." Is that all? Yes; and it is quite sufficient. Granted that he is honest and intelligent, his value is "above rubies." He knows all that is at present known; he does all that can be done. By his judgment he removes obstacles that are in the way of recovery; he gives help in the shape of well-chosen food or physic; he prevents the injurious action of ignorant friends; he inspires the patient with courage and hope; in those rare cases where specifics are available, he uses them; and by all these combined he often makes the difference between dying and living. He does not always or even often "cure" the disease, but he conducts the patient safely through it; he does not empty the ocean of its contents, but he holds the head of the feeble and fainting swimmer above the water until the shore is safely reached. That is perhaps not a function which satisfies the craving of him who is ambitious of great and wonderful things, but it is, almost certainly, the only function which the medical man will ever be called upon to perform. We do not agree with the often expressed opinion that nature has an antidote and a specific for every disease if it could only be found. Science gives little encouragement to any such view. She teaches men modesty and humility. "Patient continuance in well-doing" is her method as well as that of religion and virtue. Courage, conscience, steadfastness, and hope are the inspirations of the doctor as well as of the saint. The great principles of life are not many but few, and are the guiding stars of the individual soldiers as well as of the great army of mankind.

Our answer to the sceptic is this: "Your work is ill done if doubt and denial be the permanent condition of your mind. Doubt is good as a means but fatal as an end. If you are ever to do any practical work in the world, you must have positive principles in which you believe; they may be of the simplest and most elementary kind, but they must be there. Your faith may go no further than this, that it is a good thing for the sick man to be put to bed and to have a hot linseed poultice on. But from such a basis of conviction the right-minded and kindly-feeling medical man may develop a whole system of practice which shall acknowledge no authority but his own, and yet shall be of saving virtue to his patients. No really able man can rest in doubt as a final end. "I believe that nothing is of any value," is the "credo" of a fool. As Sir William Hamilton says, "Let us however beware that we act not the part of revolted slaves; that in asserting our liberty we do not run into licence. Philosophical doubt is not an end but a means. We doubt in order that we may believe; we begin that we may not end with doubt; we doubt once that we may believe always; we renounce authority that we may follow reason; we surrender opinion that we may obtain knowledge . . . . If the effect of philosophy were the establishment of doubt, the remedy would be worse than the disease. Doubt as a permanent state of mind would be, in fact, little better than intellectual death." Every man may be well assured that if he have no positive convictions about those things which he is daily practising, he cannot but be a miracle either of stupidity or of dishonesty.

